# Towards AI-Powered Applications: The Development of a Personalised LLM for HRI and HCI

**DOI:** 10.3390/s25072024

**Published:** 2025-03-24

**Authors:** Khashayar Ghamati, Maryam Banitalebi Dehkordi, Abolfazl Zaraki

**Affiliations:** 1 School of Physics, Engineering and Computer Science (SPECS), University of Hertfordshire, Hatfield AL10 9AB, UK; m.banitalebi@herts.ac.uk (M.B.D.); a.zaraki@herts.ac.uk (A.Z.); 2 Robotics Research Group, University of Hertfordshire, Hatfield AL10 9AB, UK

**Keywords:** large language model, human–robot interaction, human-computer interaction, personalised large language models, adaptive AI systems, AI agent

## Abstract

In this work, we propose a novel Personalised Large Language Model (PLLM) agent, designed to advance the integration and adaptation of large language models within the field of human–robot interaction and human–computer interaction. While research in this field has primarily focused on the technical deployment of LLMs, critical academic challenges persist regarding their ability to adapt dynamically to user-specific contexts and evolving environments. To address this fundamental gap, we present a methodology for personalising LLMs using domain-specific data and tests using the NeuroSense EEG dataset. By enabling the personalised data interpretation, our approach promotes conventional implementation strategies, contributing to ongoing research on AI adaptability and user-centric application. Furthermore, this study engages with the broader ethical dimensions of PLLM, critically discussing issues of generalisability and data privacy concerns in AI research. Our findings demonstrate the usability of using the PLLM in a human–robot interaction scenario in real-world settings, highlighting its applicability across diverse domains, including healthcare, education, and assistive technologies. We believe the proposed system represents a significant step towards AI adaptability and personalisation, offering substantial benefits across a range of fields.

## 1. Introduction

Decision-makers provide the crucial link between the insights derived from machine learning (ML) algorithms and their implementation in practical, real-world contexts [1,2,3,4]. Improving the accuracy of these systems allows intelligent agents to replicate human behaviour with greater fidelity. Yet, safely deploying such agents in real-world applications demands strict adherence to established guidelines, ensuring they do not pose risks to human well-being. In artificial intelligence (AI)-assisted environments, decision-making frequently involves collaboration between human agents and AI systems. The human element remains indispensable, especially in high-stakes areas such as healthcare and the legal system, where ethical considerations necessitate human oversight [5]. Recent research [6,7,8,9] further highlights various challenges faced by decision-makers in real-world scenarios, including information overload, misaligned incentives, and the ethical ramifications of algorithmic biases. Moreover, integrating AI systems into decision-making workflows calls for attention to trust, fairness, and accountability, as well as for the adoption of robust human-in-the-loop models to mitigate potential risks and ethical dilemmas [10,11,12]. These findings underscore the need for multidisciplinary approaches that bridge technical, ethical, and domain-specific expertise, enabling responsible and effective decision-making in complex contexts.

Two prominent methods for constructing such systems are Reinforcement Learning (RL) and LLMs. Although distinct in both methodology and application, these approaches offer complementary strengths. LLMs excel in tasks requiring natural language understanding and production. By contrast, RL focuses on learning optimal actions through trial and error in dynamic environments, making it particularly effective for sequential decision-making tasks such as robotics and game-playing [13,14,15]. Nevertheless, RL’s application in real-world settings often raises safety concerns, since conventional methods may fail to guarantee predictable behaviour under changing conditions. As one study notes, the inherent unpredictability of AI decisions demands the development of new safety frameworks to address uncertainties surrounding RL systems [16]. Adapting models to specific environmental conditions is consequently vital to improving agent accuracy and reliability. In this respect, the emergence of LLMs has yielded positive outcomes for robotics by enabling personalisation, allowing agents to adapt dynamically to their environment with updated data and, in turn, operate more accurately and safely.

Despite these strengths, LLMs have demonstrated limitations, particularly in scenarios that depend on human-based decision models [17,18]. As ref. [19] explains, a significant challenge arises when agents trained on static datasets fail to adapt to new circumstances, as the use of an offline (static) dataset trains model parameters on a snapshot of the data distribution, which may be misaligned with evolving real-world conditions. This issue can result in performance degradation when language distributions shift, such as the emergence of novel vocabulary, specialised domains, or changing user preferences. In contrast, online adaptation continually updates the model’s parameters with each new data point, thereby absorbing fresh linguistic patterns and maintaining relevance. Through real-time refinement, online adaptation mitigates the risk of outdated knowledge, reduces reliance on assumptions derived from historical data, and ensures the model’s understanding remains aligned with contemporary language use. Notably, personalisation constitutes a specific application of online adaptation that customises the model’s behaviour to individual users by capturing user-specific writing styles, vocabularies, and specialised knowledge. Both online adaptation and personalisation rely on incremental parameter updates and share the overarching goal of preserving model accuracy in dynamic linguistic environments.

In such instances, personalisation enables agents to make precise decisions in diverse and unfamiliar settings [20]. This capability marks a significant development across multiple applications, empowering systems to customise responses according to individual users’ preferences, objectives, and contexts. By employing user-specific data, including past interactions, interests, and linguistic patterns, LLMs can produce outputs of greater relevance and significance [21]. Techniques such as fine-tuning [22] on domain-specific datasets [23], employing retrieval-augmented generation, or utilising embeddings to encode user-specific knowledge further advance personalisation efforts. However, achieving these outcomes requires meticulous balance to ensure that personalisation remains ethically sound [24], protects user data privacy [25], avoids overfitting, and minimises biases [26]. Personalised LLMs are increasingly employed in sectors such as customer service, education, healthcare, and entertainment, offering bespoke experiences that enhance engagement and satisfaction [27,28,29].

In the present study, we examine the incorporation of LLMs as a leading approach for constructing decision-making systems. In particular, we employ the OpenAI API to utilise their LLM model in the analysis of Electroencephalography (EEG) sensor data as a case study, showcasing the effective integration of LLMs into practical applications. To accomplish this, we use Streamlit to develop an interactive chatbot interface. We further personalise the LLM agent using an EEG dataset and deploy the model on a robot, which connects to the Muse device (Muse2, InteraXon Inc., Toronto, Canada) to gather EEG data. The personalised LLM agent then interprets human emotions in a manner consistent with the conditions under which the dataset was initially collected.

The following sections will explain each of the components of this study. Section 3 discusses the concept of personalisation and the procedure we employ to personalise an LLM model using PLLM. Section 4 explains our AI agent and its components, along with their functions. Section 5 presents a case study using an EEG dataset to evaluate the agent, demonstrating its ability to process and interpret EEG data effectively.

## 2. Related Work

Uncertainty poses a significant challenge to decision-making processes in real-world scenarios. It may stem from various sources, including incomplete information, dynamic environments, and unpredictable human behaviour. Addressing these uncertainties is essential for developing robust systems capable of operating effectively in real-world contexts.

Personalisation in LLMs, a subset of online adaptation, parallels RL adaptation, as both involve refining systems to optimise their performance in specialised contexts through distinct mechanisms. In LLM personalisation, a pre-trained language model is tailored to user-specific preferences or tasks via techniques like fine-tuning or prompt optimisation [30]. Similarly, RL adaptation modifies an agent’s policy through interactions with an environment, aiming to maximise cumulative rewards via exploration and exploitation [31]. Both approaches transition from general-purpose systems to specialised applications, utilising feedback loops—user inputs or corrections in LLMs [32] and reward signals in RL. However, a key distinction lies in their learning paradigms: LLM personalisation typically relies on static datasets [33], whereas RL adaptation requires real-time interaction with the environment [31]. Despite this difference, both approaches share a conceptual overlap in fine-tuning general systems for tailored applications.

The work presented in [34] explores the reliability of using LLMs to evaluate user preferences based on personas, addressing the growing demand for LLM personalisation. The study identifies key challenges, including the limited reliability of existing LLM-as-a-Judge frameworks and the issue of persona sparsity, where simplistic persona variables provide limited predictive power. The authors propose integrating verbal uncertainty estimation to allow LLMs to express confidence levels in their judgements and implement certainty thresholding to focus on high-confidence samples, significantly improving accuracy. They further suggest that future work should address persona sparsity and explore advanced methods for uncertainty quantification, as well as extend the approach to multilingual and cross-cultural contexts. This work highlights the potential of certainty-aware LLMs as scalable solutions for personalisation tasks, especially in the absence of first-person data.

Another study introduces PersonalLLM [35], a benchmark designed to personalise LLMs to align with diverse user preferences. Unlike traditional approaches that assume uniform population-level preferences, this framework addresses the challenge of tailoring responses to individual tastes, which often vary in complex and latent ways. It employs meta-learning and in-context learning (ICL) to adapt responses for new users using limited examples. Evaluations show higher diversity and alignment with human preferences compared to baseline persona-prompting methods. However, ethical risks, such as filter bubbles and stereotyping, are acknowledged, underscoring the need for transparency and safeguards. This research lays the groundwork for advancing personalised AI while promoting ethical considerations in its deployment.

In [25], the authors proposed an end-to-end framework combining on-device LLMs with smartphone sensing to deliver personalised, context-aware services. This framework addresses privacy risks, latency, and costs associated with cloud-based LLMs by utilising lightweight, on-device models. Challenges, such as limited computational resources and occasional errors, are acknowledged, with future plans to enhance sensor diversity and model robustness. Similarly, Ref. [36] presents a framework for personalised text generation inspired by structured writing education. By employing multitask learning, the framework aligns generated text with users’ styles and contexts, demonstrating significant improvements over baselines across diverse domains. These works collectively advance the field of personalised LLMs, offering novel methodologies and addressing pressing challenges.

**Figure 1:**
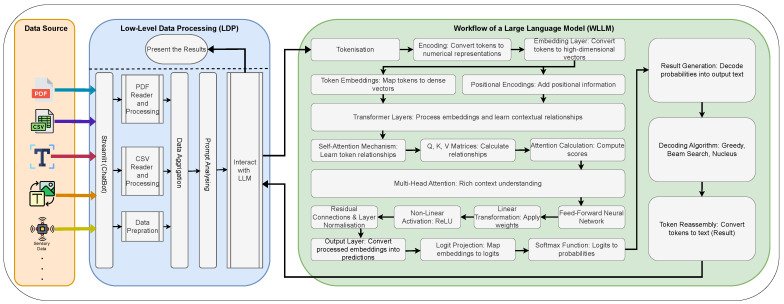
The proposed Comprehensive Guide to Integrating Large Language Models (green area [37]) with Streamlit: Developing the Personalised LLM (PLLM) Framework.

Based on our literature review, summarised in Table 1, this study proposes an AI agent for personalising large language models (PLLMs). The architecture demonstrates how an LLM agent can be integrated with other projects to utilise its advantages. Figure 1 illustrates this architecture.

## 3. AI Model Personalisation

Personalising an AI model means adapting the model to individual requirements, preferences, and contexts, thereby significantly enhancing user engagement, satisfaction, and overall outcomes [38,39]. In fact, personalisation encapsulates the notion of model adaptation, while a model continuously interacts with its environment to update its knowledge based on observational feedback to align itself with the main contextual environment [40].

LLMs have demonstrated a potential for personalisation during interaction with the environment, largely attributed to their extensive pre-trained knowledge and sophisticated fine-tuning capabilities [30,41,42]. Such ability empowers LLMs to generate contextually relevant outputs that resonate with users’ specific contexts or salient points encountered within interactions [43]. Nevertheless, an effective adaptation of LLMs post-deployment remains challenging, especially in highly dynamic environments where context rapidly evolves.

A highlighted challenge associated with model adaptation, particularly in continual learning scenarios, is the phenomenon of Catastrophic Forgetting (CF) [44,45]. CF refers to a model’s abrupt loss of previously learned knowledge when acquiring new information, primarily due to the inherent limitations of conventional gradient-based learning methods when subjected to limited, incremental data [46]. Models initially trained on large-scale datasets through extensive procedures aimed at mitigating overfitting and underfitting often find adaptation to new, typically sparse data streams challenging, resulting in compromised performance [47]. However, even considering these limitations, the targeted adaptation can considerably enhance model utility by fine-tuning outputs to reflect new environmental insights [48,49].

PLLMs draw inspiration from continual learning and personalisation paradigms [50,51], offering an effective approach for integrating new datasets into existing models without comprehensive retraining. PLLMs function as adaptive agents, assimilating new data streams as discrete tasks or knowledge sources. This integration enriches the model’s experiential knowledge, enabling nuanced reasoning about previously unseen scenarios. Unlike traditional retraining methods, PLLMs utilise specifically engineered prompts to dynamically adjust model behaviour, ensuring that new datasets are seamlessly incorporated into the model’s reasoning processes [52,53].

**Figure 2:**
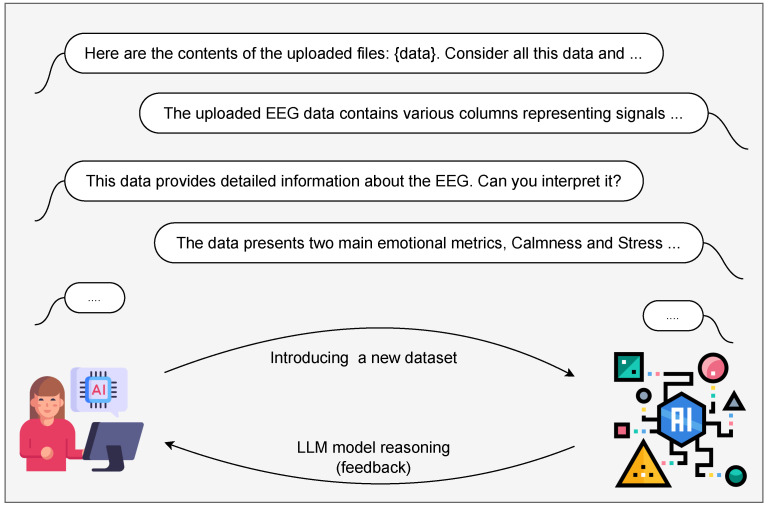
Overview of personalising data using PLLM.

Improvement based on Feedback, a foundational principle of continual learning, is a critical part of the personalisation process of PLLMs [54]. User validation, which involves interactive verification of the model’s responses, mitigates risks associated with misinterpretation and ambiguity, thereby significantly reducing the likelihood of overfitting. This interactive, user-driven feedback loop ensures the model’s newly acquired knowledge remains accurate, contextually relevant, and robust [55,56]. The process is depicted in Figure 2, illustrating dataset introduction via prompts and subsequent user-driven validation to reinforce correct data interpretation.

**Figure 3:**
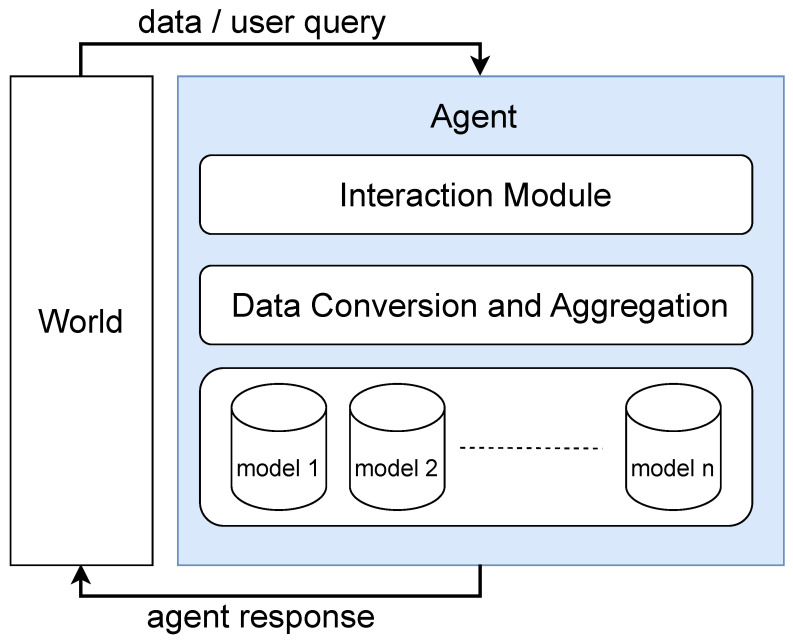
Proposed agentic AI architecture.

Moreover, PLLM architecture aligns well with emerging trends in agentic AI systems, which emphasise autonomy, adaptability, and interactive user collaboration. The conceptual architecture depicted in Figure 3 provides a comprehensive framework supporting adaptive, personalised interactions, effectively capturing the dynamic interplay between model, data, and user input.

**Figure 4:**
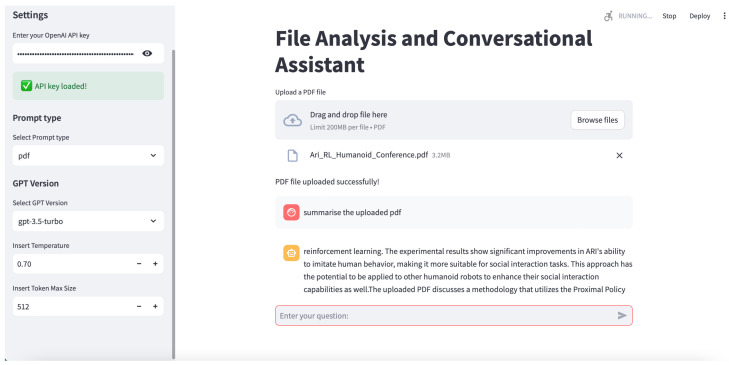
Interaction layer demonstration: Screenshot showcasing key functionalities: uploading PDF file and querying its content, securely entering API key to send prompt.

The practical efficacy of PLLMs in facilitating personalised interactions can be observed through various functionalities demonstrated in Figure 4. The illustrations highlight key capabilities, including seamless dataset integration and secure prompt processing. Collectively, these functionalities underscore the operational robustness and versatility of PLLMs in delivering personalised, contextually aligned user experiences [52,57]. The next section explains how we design the PLLM as an AI agent to personalise it using a dataset to enhance its knowledge.

## 4. AI Agent Creation

An agent can be defined as an application that attempts to achieve a goal by interacting with its environment [58]. The agent may consist of various modules, including one or more models, an interaction module for data acquisition and decision-making or action execution, and a data conversion and aggregation module for preparing the collected data. In this work, we design an agent incorporating these three key modules, as illustrated in Figure 3.

The Interaction Layer is a GUI application that gathers the required data and prompts, as well as presents completions or results. The Data Conversion and Aggregation Module is responsible for preparing data, which is then sent along with the prompts to the model. The model in this system is OpenAI, with which we interact using the respective API. The interaction layer provides a range of components that can be effectively utilised to construct a discussion between the user and the LLM model to upload the dataset and obtain feedback to personalise the model completely. This layer supports five distinct prompt types: text, image, PDF, CSV, and EEG data. Users can select one of these options to send a prompt tailored to the chosen type. In addition, it contains options for configuring the LLM model, including a text field for specifying the number of tokens. Figure 4 illustrates the GUI interaction layer.

At the core of the agent lies the model. The model serves as the primary decision-making component of the agent and should be appropriately suited to the agent’s purpose. However, it is typically not trained with specific configuration settings. However, it can be refined to enhance its familiarity with a specific topic. In subsequent sections, we will personalise the model to improve its ability to process EEG data.

A crucial parameter in model configuration is temperature, which controls the randomness of the model’s output. This parameter influences token selection by adjusting the probability distribution of possible next tokens. Lower temperatures (e.g., 0.2) produce more deterministic outputs, prioritising high-probability tokens—ideal for tasks requiring precision or factual accuracy. Conversely, higher temperatures (e.g., 1.5) encourage diversity and creativity, leading the model to generate more varied and unconventional responses. Adjusting the temperature parameter enables fine-tuning of the model’s behaviour to suit specific applications, from structured problem-solving to imaginative storytelling. User interaction occurs using the interaction layer. Based on the prompt type selected, the system sends the prompt to the model and retrieves the corresponding response.

The agent also allows users to upload a PDF or CSV file, extract its content, and query it using the OpenAI API. The file is converted into text, and to comply with token limitations, the text is segmented into manageable portions, each of which is processed by the OpenAI API to generate responses.

### 4.1. Interaction Layer

In this work, the agent utilises Streamlit to develop a chatbot user interface (UI). Using Streamlit, we design and implement a versatile chatbot UI, integrating it with an LLM model to manage a diverse range of data sources. We aim to personalise the agent to enhance its knowledge of a specific topic, which we refer to as the PLLM. Based on the prompts sent, this agent personalises the model to produce results informed by the data used to personalise it. In fact, the model will produce the results using its global knowledge by considering the data that were utilised to personalise it. This is a difference of fine-tuning which biases the model to a specific topic.

Figure 1 illustrates the architecture of this agent. Various data sources along with a prompt are transmitted to the LLM model via the Streamlit application. Depending on the data sources, the data are appropriately prepared and processed before being sent to the LLM model. The resulting outputs are then returned and presented to the user through the chatbot.

### 4.2. Data Conversion and Aggregation Module

To process the data effectively, all prompt types are converted into textual format, as the LLM operates exclusively with text-based inputs. A specific conversion method is applied according to the prompt type ensuring seamless integration into the LLM pipeline.

The subsequent step involves data aggregation. When a single prompt comprises multiple files, these are merged into a coherent input to ensure consistency and context preservation. However, as LLM models are subject to input size limitations, such as those outlined in Table 2 for OpenAI models, this constraint must be addressed. To manage this, prompts are segmented and sent in manageable chunks by the prompt analysis layer, maintaining the integrity and relevance of the input.

#### Ethical Challenges and Practical Integration of OpenAI’s Large Language Models

OpenAI’s large language models offer significant advantages in natural language processing (NLP), enhancing productivity through automation and the generation of high-quality text [30]. Their adaptability and pre-training on vast datasets make them a robust choice for projects requiring advanced language comprehension and generation [59]. However, despite these strengths, LLMs also present notable challenges and ethical considerations that must be addressed.

One of the primary concerns is bias, as these models learn from publicly available data that often contain inherent biases [26]. This can lead to the reproduction or even amplification of biased perspectives, potentially resulting in unfair or problematic outputs. OpenAI actively mitigates these risks through pre- and post-processing techniques designed to reduce bias and by curating datasets with diverse representation. Additionally, transparency initiatives, such as publishing documentation on model limitations and biases, aim to inform users of potential risks [60].

Another major ethical consideration is the misuse of generative AI. The powerful text generation capabilities of LLMs can be exploited for spreading misinformation, impersonation, or other malicious activities, raising significant ethical concerns. OpenAI addresses this issue by implementing robust content moderation systems, enforcing responsible use policies, and collaborating with external researchers and organisations to develop safety frameworks [61].

The cost and accessibility of LLMs also pose challenges, as their high computational requirements make training and deployment resource-intensive. This can create disparities in access, limiting their use primarily to well-funded organisations. OpenAI alleviates this issue by offering APIs that provide access to powerful models without requiring users to host or train them, thereby democratising access while maintaining computational efficiency [62].

Despite these challenges, OpenAI’s APIs are designed to be developer-friendly, enabling the seamless integration of advanced NLP capabilities into various applications. Unlike traditional models that require extensive fine-tuning, OpenAI’s LLMs are pre-trained on large-scale internet text, allowing them to perform a wide range of tasks effectively without additional customisation [63]. This makes them particularly suitable for research and real-world applications, as they provide state-of-the-art language comprehension while maintaining ethical safeguards [64]. However, it is important to note that while these models leverage extensive datasets, they do not retrieve real-time information during runtime, which limits their ability to provide up-to-date responses [65].

By combining technical sophistication with robust safety measures, OpenAI’s LLMs offer a powerful tool for AI-driven solutions while maintaining a commitment to ethical AI deployment. Addressing biases, preventing misuse, and ensuring equitable access remain critical factors in their responsible integration into real-world applications. Considering OpenAI’s LLM model, we will employ it to build our AI agent.

## 5. Proof of Concept for EEG Case Study

The NeuroSense dataset [66] is a novel EEG dataset collected using the cost-effective and portable Muse 2 device, which features only four electrodes. The objective of this work is to employ this dataset to personalise an LLM agent via PLLM. OpenAI’s LLM serves as the model, which undergoes personalisation by processing the dataset.

**Figure 5:**
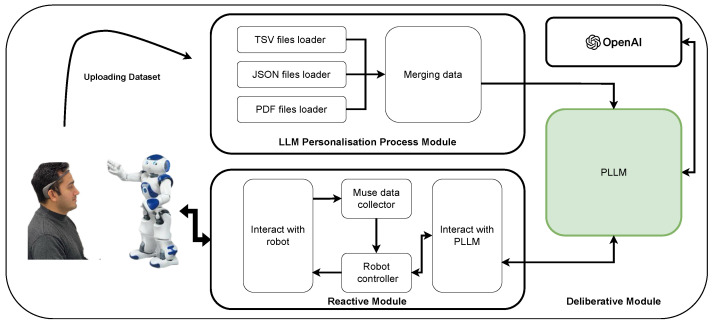
An overview of the integration of the proposed PLLM agent with EEG data.

Following this, real-time data are collected using the Muse 2 device and transmitted to the LLM to predict the participant’s emotional state. Additionally, a Nao robot is integrated with the agent to facilitate data collection from the Muse device. The PLLM processes the EEG data in collaboration with the LLM model and returns the results to the robot for presentation. Figure 5 provides an overview of this case study and its architecture.

The subsequent sections detail the implementation and integration of PLLM within the context of this case study, along with an in-depth explanation of the dataset.

### 5.1. Electroencephalography Data

Electroencephalography (EEG) is a neurophysiological monitoring method that records electrical activity in the brain. EEG signals are generated by the collective firing of neurons, particularly in the cortex, and are captured non-invasively using electrodes placed on the scalp [67]. These signals provide rich temporal insights into brain functions, such as cognitive processes, emotional states, and neural disorders, making EEG an essential tool in neuroscience, psychology, and biomedical engineering.

EEG data are characterised by their high temporal resolution and continuous time-series nature, typically segmented into frequency bands such as delta (0.5–4 Hz), theta (4–8 Hz), alpha (8–13 Hz), beta (13–30 Hz), and gamma (30–100 Hz). These bands reflect various cognitive and physiological states. For example, alpha waves are often associated with relaxation, while beta waves correlate with active thinking and attention [68]. EEG’s non-invasive nature and versatility make it a valuable resource for applications in affective computing, mental health, and human–computer interaction.

However, EEG data are inherently noisy due to artefacts from eye movements, muscle activity, and environmental interference. Advanced preprocessing techniques, including filtering, artefact removal, and segmentation, are essential to extract meaningful features from the raw signals for further analysis.

### 5.2. Personalisation of LLM Model with EEG Data

**Figure 6:**
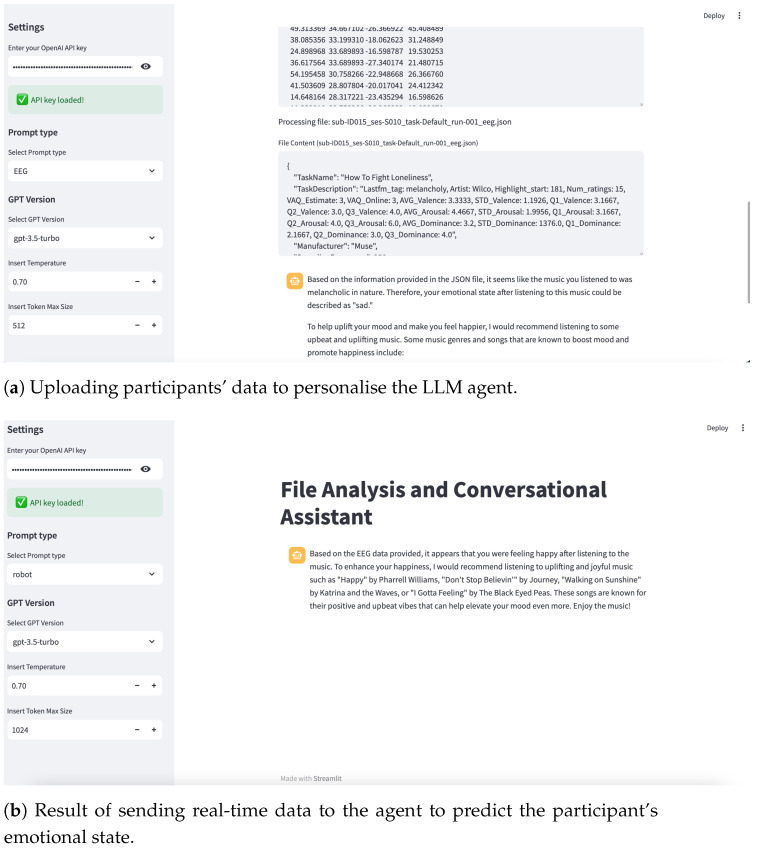
Overview of data uploading and EEG result processing using the LLM agent.

To enable real-time EEG signal processing, the LLM model is personalised using the NeuroSense dataset. This process involves introducing the dataset to the model through a structured prompt, allowing it to adapt to EEG data and refine its ability to predict emotional states. The dataset is first integrated into the model, followed by an interactive validation phase where the model’s comprehension is assessed and adjusted as necessary (see Figure 2 and Figure 6a).

Once the model has been personalised, the agent collects real-time EEG data from a Muse 2 device and transmits it to the LLM for analysis (see Figure 6b). The processed results are then relayed to a robot, which verbalises the predictions as spoken dialogue for the participant. This approach ensures that the model can effectively interpret EEG signals and respond meaningfully, demonstrating its ability to adapt to individual users’ cognitive and emotional states.

**Figure 7:**
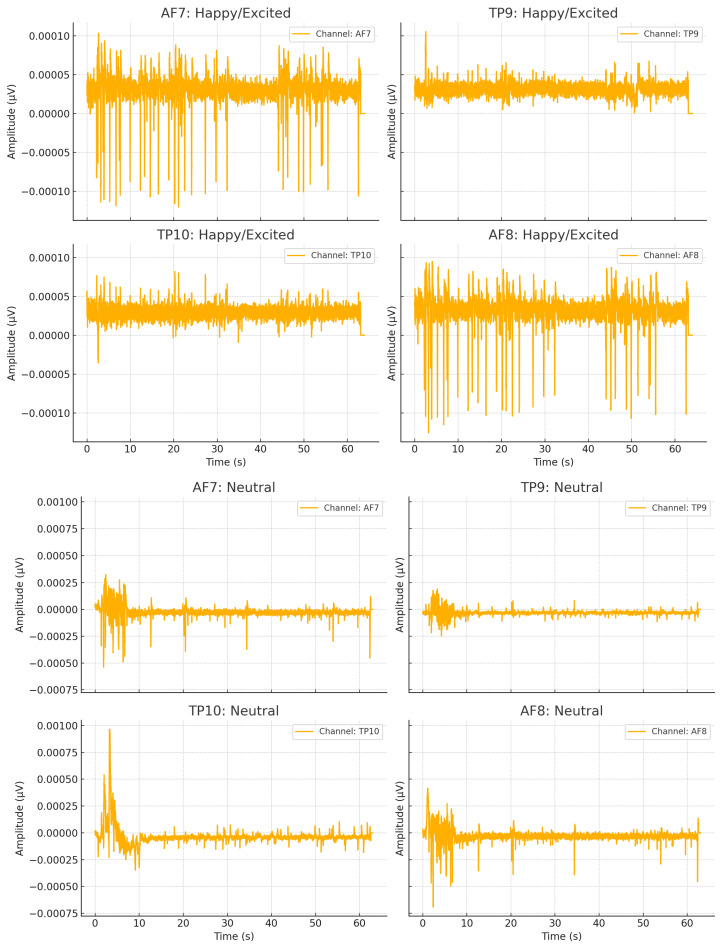
Annotating emotional state for Participants 26 and 1 using sessions 18 and 15.

To illustrate the effectiveness of this process, we examine two case studies from the dataset. Figure 7 depicts EEG data from two participants recorded while listening to different pieces of music videos. In the first case, Participant 1 was listening to Miniature Birds, while in the second case, Participant 26 was listening to Love Shack. Based on the dataset analysis, the LLM inferred that Participant 1 exhibited a Neutral emotional state, whereas Participant 26 demonstrated a Happier state after listening to their respective music selections.

To achieve this personalisation, the dataset is introduced to the model through a structured prompt, with the LLM analysing the EEG data and formulating an emotional assessment of the participant.

*Here are the contents of the uploaded files: {data}. Consider all this data and, using your knowledge, provide a single word (e.g., happy, sad, nervous, etc.) to describe how the participant was feeling emotionally after listening to this music. If the participant is not happy, suggest how you, as an assistant, could help make the participant happy. If your suggestion includes listening to music, please provide some music recommendations*.

Additionally, if the participant is not happy, the model suggests potential ways to improve their mood, including personalised music recommendations.

This structured approach ensures that the LLM is adapted to process EEG data effectively, improving its ability to provide meaningful responses and assist users based on their emotional state.

### 5.3. Real-Time EEG Signal Acquisition from Muse 2

To evaluate the performance of our agent, we integrated it with a Nao robot. The robot is responsible for collecting EEG data from the Muse 2 device, capturing a 10 s segment from a participant while they listen to music, replicating the conditions of the dataset scenario. These data are then transmitted to the PLLM, which processes the EEG signals to predict the participant’s emotional state. The robot subsequently communicates these results to the participant in real time. Figure 5 illustrates this process.

To facilitate this scenario, we extended our Streamlit application by introducing a new prompt type, referred to as *robot*. This prompt type enables the robot to establish a connection with the Muse 2 device, facilitating EEG data acquisition and its transmission to the PLLM for analysis. The Muse data collector is deployed directly on the robot, ensuring seamless communication with the Muse 2 device, real-time data retrieval, and interaction with the PLLM to process the collected information before presenting the results to the participant.

## 6. Discussion

This study introduced PLLM (Personalised Large Language Model), a novel AI agent designed for integrating LLMs with HRI and HCI applications. By leveraging personalisation techniques, the proposed approach enhances LLM adaptability, enabling models to process domain-specific data while maintaining general language understanding. As a use case, we utilised the NeuroSense EEG dataset to personalise OpenAI’s LLM, allowing it to analyse real-time EEG signals from a Muse 2 device and predict emotional states. Through this framework, we demonstrated a systematic pipeline for dataset preparation, model personalisation, real-time data processing, and interactive response generation.

A major strength of the PLLM framework is its flexibility and scalability. The architecture allows seamless integration with different data sources and projects, making it applicable beyond EEG-based affective computing. The use of OpenAI’s LLM provides a pre-trained, robust, and adaptable model, reducing the need for computationally expensive retraining. Additionally, the Streamlit-based chatbot simplifies user interaction, enabling both expert and non-expert users to personalise AI models in an intuitive interface.

Despite these advantages, several limitations and challenges remain. First, generalisation across different datasets remains an open question. While our framework effectively personalises an LLM using the NeuroSense dataset, expanding this approach to diverse datasets with varying data distributions requires further investigation. Second, the Muse 2 EEG device, while portable and cost-effective, has limited spatial resolution with only four electrodes. This constraint affects the granularity of emotional state prediction and could be improved by integrating higher-resolution EEG systems or multimodal data sources, such as facial expression analysis, voice modulation, or physiological sensors.

Another critical aspect to consider is ethics and user privacy. Personalising an LLM with EEG data introduces challenges related to data security, consent, and bias mitigation. Although OpenAI enforces content moderation policies and security measures, ensuring that AI models do not reinforce biases or generate misleading responses remains a key concern. Future work should explore techniques such as differential privacy, federated learning, and bias-correction algorithms to enhance fairness, security, and trustworthiness in AI-powered decision-making.

Furthermore, computational efficiency and cost must be considered when scaling PLLM for real-world applications. While OpenAI’s API offers state-of-the-art NLP capabilities, its reliance on cloud-based processing can be costly for large-scale deployments. Future research could explore on-device LLMs or hybrid approaches that combine local inference with cloud-based fine-tuning, ensuring a balance between efficiency, accessibility, and performance.

Overall, the PLLM framework successfully demonstrates the potential of personalised AI agents in HRI, HCI, and cognitive computing. While challenges exist, our work lays the foundation for further advancements in adaptive AI systems, emotion-aware robotics, and intelligent user interaction.

## 7. Conclusions

We devised a PLLM agent to enhance the adaptability and data interpretation in HRI and HCI scenarios. Our proposed methodology focuses on the personalisation of LLMs using the NeuroSense EEG dataset, enabling the interpretation and adaptation of the AI agent to the user-centric data dynamically. By enabling personalised data interpretation, PLLM enhances the conventional implementation strategies of LLMs, contributing to ongoing research on AI adaptability in diverse applications.

Key contributions of this work are as follows: (i) a novel approach prensented to personalise an LLM model utilising target datasets to enable adaptation and context-aware interactions without requiring full model retraining; (ii) the integration of the proposed PLLM has been achieved for a HRI scenario, where our system successfully adapted the AI agent to the target dataset in a HRI scenario; and (iii) a scalable and ethical AI personalisation framework, supporting multi-source data integration and making it applicable across diverse domains such as healthcare, education, and assistive technologies.

Future research should focus on enhancing multimodal adaptability by incorporating speech analysis, facial expression recognition, and physiological signals for a more comprehensive user adaptation.

In conclusion, we believe PLLM represents a significant step towards AI personalisation and adaptability, offering a structured approach for integrating LLMs into user-centric applications.

## Figures and Tables

**Table 1:** Key contributions in large language models.

Year	Author	Achievement
2024	Rannen-Triki et al. [19]	Proposes efficient online adaptation strategies to continually update model parameters in real time, mitigate distribution shifts, and maintain performance across evolving domains.
2024	Chen et al. [20]	Explores how LLMs enhance personalisation through interactive engagement, task expansion, tool integration, and addressing privacy challenges.
2024	Dong et al. [34]	Proposes a certainty-aware framework for predicting user preferences, achieving high accuracy and surpassing human performance.
2024	Zollo et al. [35]	Simulates user preferences via reward models, enabling ethical and scalable personalisation.
2024	Zhang et al. [25]	Combines on-device LLMs with sensing for private, real-time, and personalised services, addressing privacy risks.
2023	Rao et al. [24]	Proposes a framework for LLMs to reason ethically across diverse contexts, highlighting the need for value pluralism over fixed moral alignment.
2023	Li et al. [36]	Proposes a multistage framework enhancing LLM personalisation, achieving significant gains in domain-specific text generation.

**Table 2:** Token limits for OpenAI language models.

Model	Maximum Tokens (Response + Input)
GPT-4 (8K)	8192
GPT-4 (32K)	32,768
GPT-3.5-turbo (4K)	4096
GPT-3.5-turbo (16K)	16,384
Davinci (text-davinci-003)	4096
DeepSeek (R1)	32,768
Curie	2048
Babbage	2048
Ada	2048

## Data Availability

Data are contained within the article.
